# Capillary Microvascular Dysfunction in Rheumatoid Arthritis: The Promising Role of Nailfold Videocapillaroscopy—A Narrative Review

**DOI:** 10.3390/life16060883

**Published:** 2026-05-25

**Authors:** Elena Angeloudi, Panagiota Anyfanti, Konstantinos Tragiannidis, Eleni Korki, Eleni Aintinidou, Vasiliki Dimitriadou, Paraskevi Avgerou, George D. Kitas, Theodoros Dimitroulas

**Affiliations:** 1 Third Department of Internal Medicine, Papageorgiou Hospital, Aristotle University of Thessaloniki, 56429 Thessaloniki, Greece; elena-angeloudi@hotmail.com (E.A.); konstantinos.tragiannidis@gmail.com (K.T.); elenkourem@gmail.com (E.K.); aidinidou.el@gmail.com (E.A.); 2 Fourth Department of Internal Medicine, Hippokration Hospital, Aristotle University of Thessaloniki, 54642 Thessaloniki, Greece; arpapa61@gmail.com (V.D.); eviavgerou55@gmail.com (P.A.); dimitroul@hotmail.com (T.D.); 3 Department of Rheumatology, Russells Hall Hospital, Dudley Group NHS Foundation Trust, Dudley DY1 2HQ, UK; george.kitas@gmail.com; 4 School of Sport, Exercise and Rehabilitation Sciences, University of Birmingham, Birmingham B15 2TT, UK

**Keywords:** microcirculation, videocapillaroscopy, functional, microvascular

## Abstract

Arthritis (RA) is characterized by immune-mediated chronic inflammation and endothelial dysfunction, ultimately resulting in clinically overt cardiovascular complications. As a prototypical disease of microvascular dysfunction, RA represents an ideal model to study microvascular alterations. The dermal capillary network offers an easily accessible window to the peripheral microcirculation, whose function can be easily assessed using Nailfold videocapillaroscopy (NVC) or laser techniques. Whereas the clinical significance of structural alterations is not always clear, functional abnormalities may provide more direct insight into the dynamic status of the microvasculature and endothelial integrity. The present narrative review aims to provide an integrative overview of available studies evaluating functional abnormalities of the dermal microcirculation in RA, with particular emphasis on the emerging role of NVC as a dynamic vascular assessment tool. Several studies in RA have assessed the structure and morphology of the peripheral microvasculature using NVC, but far fewer data exist on functional alterations assessed with this method. The study of functional alterations of the dermal microvascular network in RA has largely been based on laser techniques, which consistently point towards altered microvascular reactivity. By contrast, functional NVC-related approaches remain limited, despite their potential ability to simultaneously assess structural and dynamic capillary abnormalities in vivo. Available evidence supports that NVC may be reframed as a promising functional vascular biomarker in RA. However, the available literature is characterized by small sample sizes, predominantly cross-sectional designs, and methodological heterogeneity, highlighting the need for standardized prospective studies.

## 1. Introduction

Rheumatoid arthritis (RA) is a destructive polyarthritis of autoimmune origin, affecting as many as 17.6 million people worldwide, with a trend for increasing prevalence by as much as 80% by 2050 [1]. Without appropriate treatment, RA can lead to chronic pain, varying levels of short- and long-term physical disability, poor quality of life, and premature death [1,2]. Pathophysiologically, persistent synovial inflammation and erosions of bones and cartilage, typically affecting the small joints, triggered by the increased production of autoantibodies such as rheumatoid factor and anti-cyclic citrullinated peptide antibodies, potentiate systemic inflammatory immunoresponses in RA. Thereby, clinical manifestations are not limited to the joints but may include involvement of several organs, including the cardiovascular system [3,4]. The progressive and often irreversible nature of the complications associated with the disease highlights the need for their early detection through widely available and easily applicable techniques.

More specifically, premature or accelerated atherosclerosis in the context of chronic inflammation has received increasing attention over the past decades as a major contributor to the increased cardiovascular morbidity and mortality in RA [5]. The introduction of biologics has revolutionized the treatment of RA [6]. Additional novel treatments have recently been approved, such as targeted synthetic disease-modifying anti-rheumatic drugs (DMARDs), i.e., janus kinase inhibitors, that offer the unique advantage of an oral route of administration [7,8]. Rather paradoxically, patients with RA still exhibit increased rates of atherothrombotic events, which represent a leading cause of mortality to date [5]. There is an apparent synergism between subclinical inflammation and traditional cardiovascular risk factors in RA, which remain highly prevalent and largely undertreated [9]. Failure to detect subclinical cardiovascular disease at an early stage, before the establishment of overt cardiovascular manifestations, may at least partially account for the increased burden of cardiovascular disease in RA.

For the early detection of subclinical cardiovascular disease, novel imaging tools, such as coronary computed tomography angiography, cardiac positron emission tomography, and cardiac magnetic resonance imaging, may be implemented. However, such instruments have not been widely implemented in clinical practice due to their cost, availability, or even radiation exposure [10,11]. On the other hand, direct, real-time, dynamic visualization of the microvasculature is feasible with other methods such as nailfold videocapillaroscopy (NVC), which provides information on both morphological (structural) and dynamic (functional) features of the microcirculation [12]. Other methods, such as laser Doppler techniques, offer a dynamic assessment of the microvasculature but are merely used for research purposes [13].

The dermal capillary network offers an easily accessible window into the peripheral microcirculation, whose function can be easily assessed using NVC or laser-based techniques. To date, most studies evaluating microvascular dysfunction in patients with RA have focused on morphological alterations of the microvasculature, whose clinical significance remains uncertain [14]. Although the dynamic assessment of the microvasculature may provide valuable information on functional abnormalities, far fewer studies have assessed functional alterations of the microvasculature in RA. As a result, much of the currently available evidence regarding functional dermal microvascular dysfunction in RA originates from laser-based techniques, whereas NVC-related functional approaches remain comparatively limited but increasingly promising due to their ability to simultaneously assess structural and dynamic capillary abnormalities in vivo. Therefore, the purpose of this narrative review was to provide an overview of clinical studies that have assessed functional alterations of the dermal microcirculation in patients with RA, while summarizing observed associations with disease-related parameters or cardiovascular risk factors. Special emphasis will be placed on the contribution of NVC, as this method is probably the most “mature” for dynamic visualization of the microcirculation in RA based on its wide availability, low cost, easy implementation, and high repeatability.

## 2. Literature Search Strategy

This manuscript was designed as a narrative review. A literature search was performed in the PubMed database to identify relevant articles published in English up to February 2026. Observational and interventional studies evaluating functional alterations of the dermal microcirculation in adult patients with RA were considered eligible. Studies were selected based on thematic relevance to the scope of the review, while additional relevant publications were identified through manual screening of reference lists. Search terms included combinations of the following keywords: “rheumatoid arthritis”, “microvascular”, “microcirculation”, “functional”, “nailfold capillaroscopy”, “videocapillaroscopy”, “laser Doppler”, and “endothelial dysfunction”.

## 3. RA as a Prototypical Disease for the Study of Microvascular Endothelial Dysfunction

Even after accounting for traditional cardiovascular risk factors such as smoking, dyslipidemia, or hypertension, patients with RA experience cardiovascular events more frequently than expected. This persistent gap has shifted scientific interest toward disease-related mechanisms, with RA itself now being regarded as a condition that intrinsically promotes vascular risk [5].

### 3.1. Endothelial Dysfunction in RA: The Interplay Between Inflammation and Autoimmunity

Among the proposed pathways, endothelial dysfunction has emerged as a major driver of cardiovascular morbidity and mortality in RA [15]. While several studies have demonstrated structural and functional impairment of the large vessels, for example, increased arterial stiffness and carotid atherosclerosis [16,17], it has become increasingly apparent that the problem is not restricted to the macrocirculation but also involves the microvasculature, which is not typically assessed in clinical practice. Due to its size and intimate contact with the immune system, the microvascular bed may be among the earliest sites of damage, while the “cross-talk” between the small and large arteries may further perpetuate microvascular endothelial dysfunction in the context of systemic diseases such as RA [18,19].

The endothelium is an active regulator of vascular homeostasis, participating in vasomotor control, coagulation, leukocyte recruitment, and inflammatory signaling [20]. Under the persistent inflammatory burden that characterizes RA, this regulatory capacity becomes impaired, favoring vasoconstriction, thrombosis and immune cell adhesion. Reduced nitric oxide availability, oxidative stress, and increased expression of adhesion molecules are typical manifestations of this altered endothelial phenotype [21]. Continuous exposure to inflammatory mediators further promotes endothelial activation and dysfunction. Cytokines, including TNF-α, IL-1β and IL-6, induce the expression of adhesion molecules such as VCAM-1, ICAM-1, and E-selectin, facilitating leucocyte adhesion and migration into tissues [22]. These mechanisms are particularly relevant in the microcirculation, especially at the level of post-capillary venules, where immune cell extravasation predominantly occurs [23].

The effects of chronic inflammation on the microcirculation are further potentiated by autoimmunity, which further fuels vascular injury. Seropositivity for rheumatoid factor or anti-citrullinated protein antibodies has been associated with a worse cardiovascular profile and with evidence of endothelial impairment [24,25]. Immune complexes can deposit on or interact with the endothelial surface, activate the complement system, and promote local inflammation [26]. Within the extensive microvascular network, the cumulative impact of these mechanisms may become clinically significant.

### 3.2. Microvascular Endothelial Dysfunction in RA

A considerable body of clinical evidence supports the presence of microvascular endothelial impairment in RA. Abnormal responses have been described in the cutaneous circulation, in peripheral resistance vessels and within the coronary microcirculation, and have been extensively reviewed elsewhere [21]. Importantly, such abnormalities can be observed even in patients without overt cardiovascular manifestations [27,28]. Even when functional tests appear normal in very early disease, biomarkers indicating endothelial activation are often elevated, suggesting that structural or molecular injury may precede detectable physiological impairment [21,29]. Notably, such alterations do not always correlate with markers of disease activity or standard disease characteristics. This suggests that vascular injury may reflect the cumulative inflammatory burden over time rather than only the current inflammatory state. Specific cytokines, however, appear more closely associated with vascular abnormalities. Associations between TNF-α or IL-1β levels and impaired endothelial responses have been previously reported, supporting a more direct pathogenic role for particular inflammatory pathways [30].

Recognition of microvascular involvement also helps interpret several clinical observations and could potentially improve cardiovascular risk stratification by identifying high-risk patients. Remarkably, patients with RA are less likely to exhibit typical signs and symptoms of cardiac involvement and hence, more likely to remain undiagnosed [31]. Unveiling microvascular dysfunction may be even more meaningful in the absence of accurate cardiovascular risk classification in RA. To date, all cardiovascular risk calculators, either generic or disease-adapted, have failed to provide an accurate estimation of cardiovascular disease risk when applied in large, real-life RA cohorts [32].

**Figure 1:**
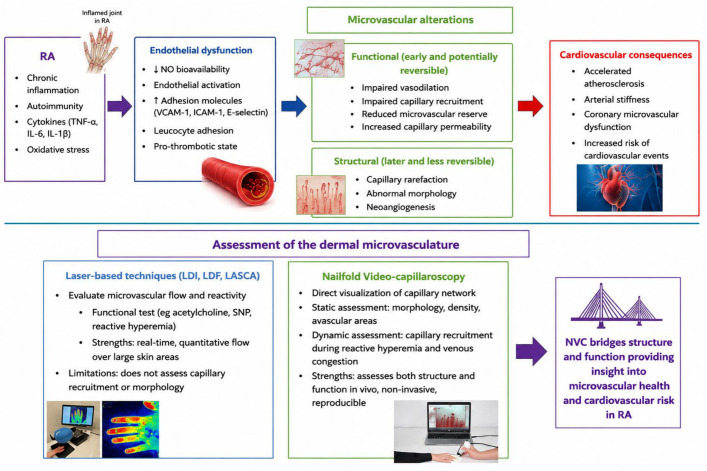
Conceptual framework of microvascular dysfunction in RA and the complementary role of NVC. Chronic inflammation, oxidative stress, and autoimmune activation in RA contribute to endothelial dysfunction, characterized by impaired nitric oxide bioavailability, endothelial activation, and increased leukocyte adhesion. These processes promote both functional and structural alterations of the dermal microcirculation. Functional abnormalities include impaired vasodilation, reduced capillary recruitment, abnormal vascular reactivity, and reduced microvascular reserve, whereas structural abnormalities include capillary rarefaction and morphological capillary alterations. Progressive microvascular impairment may contribute to arterial stiffness, accelerated atherosclerosis, coronary microvascular dysfunction, and increased cardiovascular risk in RA. Laser-based techniques primarily assess microvascular flow and vascular reactivity, while NVC enables direct in vivo visualization of the capillary network and simultaneous evaluation of both structural and dynamic functional abnormalities. Dynamic NVC approaches, particularly when combined with reactive hyperemia or venous congestion protocols, may provide insight into impaired capillary recruitment and functional microvascular reserve. Upward arrow (↑) indicates increased expression, activation, or levels, whereas downward arrow (↓) indicates reduced bioavailability. Abbreviations: RA, rheumatoid arthritis; NVC, nailfold videocapillaroscopy; NO, nitric oxide; VCAM-1, vascular cell adhesion molecule 1; ICAM-1, intercellular adhesion molecule 1; TNF-α, tumor necrosis factor alpha; IL-6, interleukin 6; IL-1β, interleukin 1 beta; LDI, laser Doppler imaging; LDF, laser Doppler flowmetry; LASCA, laser speckle contrast analysis; SNP, sodium nitroprusside.

Collectively, RA represents a prototype human model in which inflammation, autoantibodies, and vascular pathology coexist. A conceptual overview of the proposed mechanisms linking chronic inflammation, endothelial dysfunction, microvascular abnormalities, and cardiovascular risk in RA, as well as the complementary role of NVC and laser-based techniques, is presented in Figure 1. The study of how these elements interact has reinforced the notion that the microcirculation is particularly vulnerable and is often affected early, sometimes irrespective of the level of apparent clinical activity. Early identification of microvascular dysfunction in RA may ultimately refine cardiovascular risk assessment and guide the development of preventive approaches tailored to this population.

## 4. Functional Alterations of the Dermal Microcirculation in RA: Observations with Laser Doppler Imaging Techniques

The nailfold dermis is an easily examined site due to the morphology of its capillaries (straight and parallel major axis capillaries), frequently affected by systemic disease. The study of functional abnormalities of cutaneous microvascular flow in RA has been extensively based on optical techniques for assessing blood flow, such as Laser Doppler imaging (LDI), Laser Doppler flowmetry (LDF) or Laser Speckle Contrast Analysis (LASCA), which offers advantages in terms of improved spatial and temporal reproducibility, particularly when coupled with reactivity tests as compared with conventional LDF [33,34]. Laser methods evaluate microvascular flow over a large skin surface area and can be applied in real time, making it particularly appropriate for studying microcirculatory reactivity after implementation of functional tests as compared to resting conditions. Microvascular reactivity can be assessed by applying various microvascular tests that explore distinct physiological and pathophysiological pathways, all implicated in the control of skin microvascular function. Among them, iontophoresis with vasoactive drugs such as acetylcholine and sodium nitroprusside, post-occlusive reactive hyperemia (PORH), and local thermal hyperemia are widely used [35,36].

Studies in RA evaluating functional alterations of the dermal microvasculature using Laser Doppler techniques are summarized in Table 1. The majority were cross-sectional studies with many using a control group as a comparator. Only three studies included a longitudinal design.

In this context, it has been evidenced that patients with RA exhibit both reduced capillary flow and impaired reactive hyperemia, which suggests reduced microcirculatory reserve and limited vasodilatory capacity of the skin microvascular network [38]. A recent study conducted in patients with RA without cardiovascular comorbidities supports that skin microcirculation dynamics are significantly impaired compared to healthy controls, both under baseline conditions and during functional stimuli [28]. This leads to early microvascular impairment independent of the presence of classic cardiovascular risk factors. The authors highlighted that the characteristics of the study population (longstanding disease with low disease activity and low systemic inflammatory load at the time of the examination) denote that impairment of microvascular function reflects the cumulative effects of systemic inflammation on vascular architecture during the course of the disease.

Notably, improvements in microvascular endothelial function have been reported in patients with RA following treatment with biologics. In a prospective, longitudinal study, improved microvascular endothelial response was observed following a 12-week course with adalimumab [39], and the same has been observed with tocilizumab [46]. In a pilot study by Datta et al. [37], skin microvascular function in patients with active RA was found to be significantly impaired compared with healthy controls, while anti-inflammatory treatment was associated with improvement in functional indices, with statistically significant differences both compared with controls and after treatment (both *p* < 0.00001). Microvascular reactivity was assessed non-invasively using Laser Doppler imaging, in combination with acetylcholine iontophoresis to assess endothelium-dependent vasodilation and sodium nitroprusside to assess endothelium-independent response in the forearm region. A similar functional assessment by Galarraga et al. [49] studied 51 patients with RA without a history of cardiovascular disease, who used Laser Doppler perfusion combined with iontophoresis of acetylcholine and sodium nitroprusside to assess endothelium-dependent and endothelium-independent microvascular responses. Patients who responded clinically to therapy (*n* = 31) demonstrated notable improvements in both endothelium-dependent and endothelium-independent vasodilatory responses. This finding reinforces the interpretation that systemic inflammation is directly related to dysfunctional vasoreactivity at the microvascular level. Using patients with osteoarthritis as a control group, reduced peak capillary blood cell velocity was observed in RA patients [50]. Neither capillary blood cell velocity during rest nor during reactive hyperemia was correlated with the levels of soluble adhesion molecules, while no differences were observed in resting or peak capillary blood cell velocity between RA patients with or without extraarticular manifestations.

Additionally, other studies based on Laser Doppler techniques have reported microvascular abnormalities in RA in conjunction with inflammatory activation markers [38,45]. However, the association between altered microvascular function and inflammatory burden was not unanimously reproduced in relevant studies [40,41,43], and cardiovascular risk factors such as a sedentary lifestyle have been correlated with microvascular endothelial-dependent function [47]. This finding is consistent with available literature from the majority of cross-sectional and longitudinal studies, which have shown a strong impact of cardiovascular risk factors but inconsistent associations between disease-related inflammation and the vasculature [51]. Further studies by Sandoo and colleagues using Laser Doppler techniques have confirmed microvascular endothelial dysfunction, also supporting the concept of generalized vascular impairment in RA [42,44].

Overall, these heterogeneous findings likely reflect important differences in study populations and methodology. Studies including patients with early RA or low current inflammatory burden may demonstrate relatively preserved microvascular responses, whereas cohorts with longstanding disease may reflect cumulative vascular injury despite clinically controlled disease activity. In parallel, treatment exposure, particularly biologic therapy, may partially reverse functional abnormalities and thereby modify cross-sectional associations with inflammatory markers. Methodological variability may also contribute to discrepant results, as Laser Doppler studies have used different platforms (LDI, LDF, and LASCA), vascular territories, and reactivity protocols (iontophoresis, PORH, and thermal testing), each interrogating distinct aspects of endothelial and microvascular function. In addition, most available studies were cross-sectional and included relatively small patient populations, limiting the ability to establish causal relationships or determine the prognostic significance of the observed microvascular abnormalities. The limited number of longitudinal studies further restricts understanding of the temporal evolution and reversibility of functional microvascular dysfunction in RA. Therefore, inconsistency across studies should not necessarily be interpreted as contradictory evidence, but rather as the consequence of biological and technical heterogeneity.

Collectively, the use of Laser Doppler imaging techniques for the direct visualization of the microvasculature is emerging as a reliable tool for studying microvascular reactivity in populations at increased cardiovascular risk, including patients with RA. However, it should be noted that these techniques mainly reflect flow changes rather than actual capillary recruitment, which limits the ability to distinguish between functional hypoperfusion and structural capillary rarefaction. From this scope of view, LDI data in RA provide strong evidence for the existence of functional microvascular dysfunction, but do not allow direct quantification of functional capillary density, highlighting the complementary value of NVC in reactive hyperemia and venous congestion protocols. Furthermore, cost and availability issues remain major barriers to the wide implementation of this technique in patients with systemic diseases such as RA.

## 5. NVC as a Direct Window to the Dermal Microcirculation

- Technical principles and methodology

Following Maurice Raynaud’s observations in the 19th century that several capillary abnormalities correlate with clinical findings, NVC was introduced by Maricq and Lobard as early as 1912 in the rheumatology field for the early detection of capillaroscopic patterns associated with scleroderma [52]. NVC is a non-invasive, two-dimensional imaging technique that has recently come into the limelight as an indicator of peripheral vasculopathy in the context of systemic diseases [53].

More specifically, the dermal capillary network is a standard site of assessment with NVC. A typical examination protocol involves capillary assessments with patients in a sitting position, with their hands positioned at heart height. Prior to the examination, patients should adapt for a few minutes to room temperature (20–22 °C) to avoid vasoconstriction of nailfold circulation. Fingers with trauma or infection should be avoided for the probability of confounding results (hemorrhages or neo-angiogenesis). Immersion oil is applied to the skin surface to improve image quality by enhancing the epithelium transparency. NVC consists of a probe with a low magnification lens (×20) for a whole view of the nailfold capillary system, or a high-magnification lens (×200) for a more detailed study of the circulation and each capillary alone. Instruments include an image analysis software enabling the storage of images and their future usage. NVC allows for both static assessments in obtained images and dynamic blood flow assessment of microvasculature following appropriate stimuli [14,54].

- Static vs. dynamic assessment

NVC assessments can be divided into two components: static and dynamic. During static assessment, the morphological-structural state is studied. A panoramic view of the image gives a qualitative point of the architecture, the density, and the dimensions of the capillaries, whereas the measurements of some variables of each capillary per linear millimeter provide a quantitative measure. Structural assessment includes evaluation of capillary morphology, organization, density, and size, as well as microhemorrhages, avascular areas, and neo-angiogenesis. Morphological abnormalities detected by means of NVC in patients with RA have been recently reviewed [14]. Most available studies have mainly documented non-specific morphological alterations in RA, although the exact patterns and their clinical significance remain a subject of ongoing research. For instance, dermal capillary rarefaction has been previously described in RA [12], but the static assessment of capillary density cannot discriminate whether capillary rarefaction truly represents an abnormality with functional implications or simply corresponds to underperfused capillaries that may be recruited under appropriate stimuli [52].

**Figure 2:**
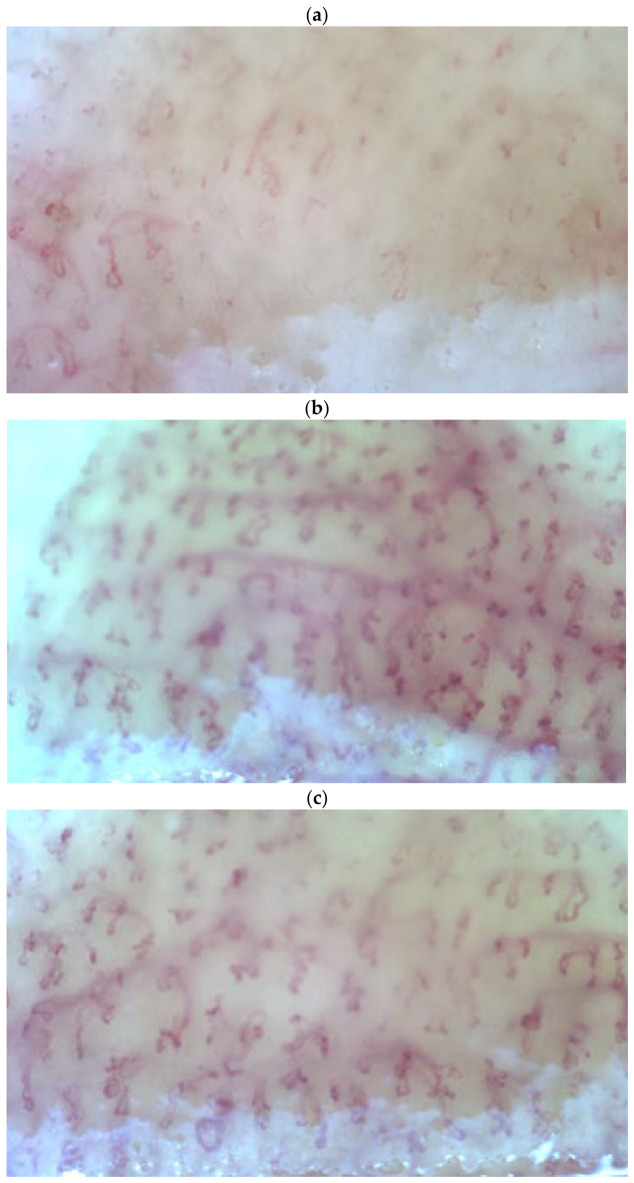
Nailfold videocapillaroscopy (NVC) images demonstrating functional microvascular assessment in a patient with rheumatoid arthritis: (**a**) at baseline, (**b**) during reactive hyperemia, and (**c**) during venous congestion, in the dorsum of the fourth finger. Capillary density at baseline was defined as the number of continuously erythrocyte-perfused capillaries per optical field (1 mm^2^) recorded over a 15 s period. Post-occlusive reactive hyperemia was induced by inflating a small finger cuff to suprasystolic pressure (260 mmHg) for 4 min. Immediately after cuff release, all continuously or intermittently perfused capillaries were recorded for 15 s to assess hyperemic capillary recruitment. Venous congestion was induced by inflating the cuff to 60 mmHg for 2 min, followed by a 15 s recording of perfused capillaries to evaluate capillary recruitment under increased venous pressure.

Functional alterations of the bloodstream are observed during dynamic assessments. Although less literature is available concerning functional parameters, typical functional assessments include capillary blood flow, low-temperature-induced vasoconstriction, hyperemia, and capillary wall integrity. In dynamic assessment, blood flow, perfusion, capillary filling, and real-time red blood flow movement can be noticed [14,54]. Functional assessment of the microvasculature requires implementation of a stimulus to evaluate dermal microvascular responses such as reactive hyperemia. More specifically, reactive hyperemia is a well-studied physiological response characterized by a transient increase in blood flow following a period of ischemia, reflecting the functional integrity of microcirculation. Experimentally, this response is usually elicited by transient occlusion of a limb artery with a tourniquet (PORH), although alternative methods, such as passive limb elevation, have also been described as less unpleasant for the subject [55,56]. During the release of the occlusion, the sudden increase in flow leads to vasodilation and increased local perfusion, a condition that is a combined result of hemodynamic and local vasoactive mechanisms (Figure 2).

## 6. Functional Alterations of the Dermal Capillary Network in RA: The Emerging Role of NVC

Nailfold videocapillaroscopy (NVC) offers a unique window to study microvascular structure and function non-invasively and in vivo. Originally introduced as a bedside tool to facilitate early diagnosis of rheumatic diseases, the emerging role of this technique as an early indicator of subclinical vascular alterations has received increasing recognition in the cardiovascular field. NVC has the potential of identifying both structural (morphological) and functional alterations of the nailfold capillary network. Most previous studies using NVC in patients with RA were dedicated to the description of morphological (structural) abnormalities, with only a limited number offering information for functional assessments. This relative lack of functional NVC studies in RA may be partially explained by the historical use of capillaroscopy primarily as a morphological diagnostic tool in rheumatology, particularly in systemic sclerosis and related connective tissue diseases [54,57,58]. In addition, dynamic NVC-related protocols remain less standardized and technically less established compared with laser-based techniques, which have traditionally dominated the field of functional microvascular assessment due to their ability to continuously quantify blood flow responses during vascular reactivity testing [59,60,61]. Available studies evaluating functional abnormalities of the dermal microvascular network by use of NVC in patients with RA are presented in Table 2.

In a study published more than three decades ago, functional damage to the capillary wall was reported after the intravenous injection of fluorescein sodium using dynamic fluorescence nailfold NVC in patients with RA [62]. Specifically, Hachulla et al. demonstrated increased transcapillary dye diffusion not only in patients with systemic rheumatoid vasculitis, but also in a substantial proportion of patients with active RA without overt vasculitis, suggesting the presence of subclinical microvascular endothelial injury. Interestingly, conventional capillaroscopy failed to differentiate between these patient groups, whereas dynamic fluorescence videomicroscopy was able to detect functional alterations of capillary permeability. These findings support the concept that NVC-based dynamic approaches may provide functional information beyond conventional morphological assessment and may contribute to the evaluation of microvascular dysfunction in RA. Other microvascular alterations pointing towards impaired microvascular blood flow have thereafter been described in RA. Nevertheless, studies using NVC for the evaluation of functional abnormalities of the dermal capillary network remain extremely limited to date. In addition, reduced capillary blood flow and cold-induced vasospasm have also been observed in RA using NVC in combination with a cold challenge test, consistent with vascular dysregulation in RA [63]. In the study by Pache et al., patients with RA exhibited significantly elevated plasma endothelin-1 levels along with reduced capillary blood flow and frequent cold-induced capillary blood standstill, further supporting the presence of impaired microvascular reactivity in RA. Given the vasoconstrictive and endothelial effects of endothelin-1, these findings suggest a potential link between endothelial dysfunction and abnormal dermal microvascular regulation in this population.

The most recent evidence of impaired microcirculatory responses detected with NVC comes from the study by Caraba et al. [64]. Caraba et al. showed that patients with active RA exhibited higher inflammatory load and lower capillary density compared to controls in all phases of NVC: in the baseline state (30.08 ± 1.70 versus 35.11 ± 1.71 capillaries/mm^2^), during PORH (32.30 ± 2.27 versus 40.81 ± 1.98 capillaries/mm^2^) and after venous congestion (34.57 ± 2.41 versus 42.78 ± 2.22 capillaries/mm^2^ (all *p* < 0.0001) [64]. Importantly, reduced capillary density during PORH reflected impaired functional capillary recruitment and reduced microvascular reserve, whereas reduced capillary density after venous congestion suggested the presence of structural capillary rarefaction. Moreover, capillary density indices were significantly associated with both DAS28 and TNF-α levels, further supporting the link between systemic inflammation and microvascular endothelial dysfunction in RA. After 12 months of treatment with methotrexate and TNF-α inhibitors, remission was achieved with a parallel improvement in capillary density (baseline phase 32.84 ± 1.91, PORH 35.41 ± 2.19, and venous congestion phase 39.20 ± 2.80 capillaries/mm^2^, all *p* < 0.0001), but without complete normalization compared with the control values. The partial reversibility of these abnormalities following anti-inflammatory treatment supports the concept that at least part of the microvascular impairment in RA is dynamic and potentially amenable to therapeutic modulation.

Collectively, the available evidence suggests that NVC may provide valuable insight not only into structural microangiopathic abnormalities but also into functional alterations of the dermal microcirculation in RA. Dynamic NVC-based approaches appear capable of detecting impaired capillary recruitment, abnormal vascular reactivity, and altered microvascular reserve, even in the absence of overt cardiovascular disease. Although currently available data remain limited and heterogeneous, these findings support the emerging role of NVC as a non-invasive tool for the assessment of microvascular endothelial dysfunction and possibly early cardiovascular risk stratification in RA. Importantly, the predominance of cross-sectional designs and the absence of longitudinal cardiovascular outcome data currently prevent definitive conclusions regarding the prognostic value and clinical applicability of functional NVC abnormalities in RA. Further longitudinal studies are warranted to better define the prognostic and clinical implications of functional NVC abnormalities in this population.

- Evidence derived from other disease states

Although relevant studies remain limited in RA, available evidence from other disease states suggests promising potential for the dynamic evaluation of the dermal microvascular network using NVC. Studies that have used NVC coupled with PORH for the functional assessment of skin microcirculation mainly originate from the field of arterial hypertension, where the concept of capillary rarefaction (morphological and/or functional) has been principally studied as a contributing mechanism to the pathogenesis of hypertension per se. Already in the classic works of Antonios et al. and Serné et al. [65,66], it has been stated that reduced capillary density in AH is not a homogeneous condition, but reflects both early primary structural thinning of the capillary network as well as functional inability to completely fill and recruit existing capillaries. More specifically, the application of functional tests such as post-occlusive hyperemia and venous congestion has shown that a significant part of the reduction in functional capillary density is a result of the incomplete recruitment of capillaries with intermittent perfusion, beyond the pure anatomical absence of vessels [65,66].

Within the same methodological context, the study by Penna et al. [67] is of particular importance and can be considered a model for the current approach. Functional capillary density was sequentially evaluated in the baseline state, during PORH, and under conditions of venous congestion. Patients treated for idiopathic hypertension systematically presented lower capillary density in all stages compared to normotensive controls, both at rest, as well as during PORH and venous congestion, with all differences being statistically significant (*p* < 0.01). Based on the capillary density during venous congestion, which is considered a condition of maximal enhancement of the available capillary network, the authors estimated a structural deficit of ~25%, concluding that the observed desertification of the microcirculation in arterial hypertension results from a combination of stable structural alterations and dynamic functional disorders of capillary filling and recruitment.

Similar observations of dynamic microvascular impairment using NVC coupled with PORH have been reported in other diseases characterized by increased cardiovascular risk. This reinforces the generalizability of the functional capillary recruitment model. In patients with type 2 diabetes, reduced capillary density at rest and insufficient increase after ischemic stimulation appeared to be attributed to a combination of endothelial dysfunction and reduced vasodilatory reserve [68]. In patients with stage 2–4 chronic kidney disease whose structural and functional capillary reservoir was assessed with NVC [69], capillary density showed a progressive decrease with the deterioration of renal function already at baseline. In addition, functional capillary density decreased during PORH, and the same was observed under conditions of venous congestion. By contrast, markers of macrovascular impairment, such as pulse wave velocity and carotid intima-media thickness, showed numerically higher values that did not reach statistical significance, suggesting that microvascular dysfunction may evolve more rapidly than macrovascular lesions in the progression of chronic kidney disease. Overall, these data document that both structural and functional capillary rarefaction progressively worsen with loss of renal function and may contribute substantially to the increased cardiovascular burden in patients with chronic kidney disease.

Similarly, in individuals with obesity and metabolic syndrome, an attenuated post-occlusive increase in functional capillary density has been described, even in the absence of clear anatomical thinning, suggesting that functional microvascular dysfunction may precede the establishment of permanent morphological alterations [70,71]. These findings support that PORH is a sensitive tool for detecting early disorders of microcirculatory reserve before the full manifestation of morphological vascular rarefaction.

## 7. Conclusions and Future Directions

RA is associated with accelerated atherosclerosis, resulting in increased cardiovascular risk. Microvascular endothelial dysfunction may be observed early in the course of the disease, even in the absence of overt cardiovascular manifestations. The dermal capillary network offers a unique window for the in vivo study of the peripheral microcirculation, easily accessible with non-invasive methods. Several studies have described divergent morphological alterations of the cutaneous microvasculature, yet their clinical significance often remains undetermined. Functional assessment of such alterations in RA has been largely based on Laser Doppler techniques, such as LDI/LDF or LASCA, that apply various microvascular tests for the study of microvascular reactivity. NVC is a convenient, easily reproducible method, widely used in rheumatology, that allows for both static and dynamic blood flow assessments following appropriate stimuli. Importantly, compared with laser-based techniques that primarily assess flow-related parameters, NVC offers the additional advantage of direct in vivo visualization of the capillary network, enabling the simultaneous evaluation of structural and functional microvascular abnormalities. Accordingly, laser-based techniques and NVC should probably be viewed as complementary rather than interchangeable approaches, as laser methods primarily quantify perfusion-related flow responses, whereas NVC additionally enables direct visualization of capillary morphology, density and recruitment in vivo. Dynamic NVC-related approaches may therefore provide insight into different stages of microvascular involvement in RA, ranging from early functional vascular dysregulation and impaired capillary recruitment to more advanced structural capillary rarefaction.

Although still an area of research, the potential clinical implications of NVC have recently expanded to cover a wide range of systemic diseases characterized by vasculopathy. Nevertheless, only a limited number of studies have applied NVC for the functional assessment of the dermal capillary network in RA. Still, available data with either method consistently point towards altered microcirculatory function, as assessed in the cutaneous microvasculature, in the context of RA. Such alterations often coincide with inflammatory markers, although they are more likely to reflect the cumulative burden of the disease rather than the current inflammatory activity.

Taken together, the currently available evidence supports the concept that NVC may represent a promising adjunctive tool for the evaluation of subclinical microvascular dysfunction in RA and potentially a bridge between chronic inflammation, endothelial dysfunction, and cardiovascular risk. In this context, functional NVC abnormalities may not simply reflect isolated peripheral vascular findings, but rather be part of a broader systemic microvascular phenotype associated with RA.

Nevertheless, interpretation of the currently available literature should be performed with caution. Existing studies evaluating functional microvascular abnormalities in RA using NVC remain limited in number and are characterized by substantial methodological heterogeneity, including differences in patient populations, disease duration, inflammatory burden, vascular protocols, and assessed capillaroscopic parameters. In addition, most available studies are cross-sectional and include relatively small sample sizes, limiting the ability to establish causal associations or determine the prognostic significance of observed abnormalities. The absence of standardized dynamic NVC protocols further complicates comparisons across studies and currently limits broader clinical applicability.

Long-term, adequately powered prospective follow-up studies are needed to determine the exact nature of the microcirculatory alterations observed in RA and their potential prognostic significance with respect to the clinical course of the disease and the development of subsequent cardiovascular manifestations. Future studies should also focus on the standardization of dynamic NVC protocols, the identification of clinically meaningful dynamic capillary indices, and the integration of NVC findings with established markers of vascular dysfunction and cardiovascular risk stratification models.

## Figures and Tables

**Table 1:** Studies evaluating functional dermal microvascular abnormalities in RA using Laser Doppler techniques.

Study & Year of Publication	Type of Study	Study Population	Method of Microvascular Assessment	Key Findings
Datta et al., 2007 [37]	Clinical trial, pilot study	In total, 8 RA patients and 8 healthy controls	Laser Doppler imaging with iontophoresis	Impaired microvascular vasodilatory responses in RA; improvement after anti-inflammatory treatment; and associated with CRP and pain scores.
Arosio et al., 2007 [38]	Cross-sectional	In total, 65 women with RA and 40 healthy women	Laser Doppler flowmetry	Impaired microcirculatory reactivity, endothelial dysfunction and increased arterial stiffness in RA; association with CRP.
Foster et al., 2010 [39]	Cross-sectional	In total, 66 patients with RA, 48 community controls, and 25 disease controls	Laser Doppler imaging + iontophoresis	Abnormal endothelial-independent-vasoreactivity associated with inflammatory markers (CRP, ESR, and vWF); no vascular improvement after 2–4 weeks.
Van Eijk et al., 2011 [40]	Cross-sectional	In total, 15 RA patients and 15 matched controls	Laser Doppler fluxmetry with iontophoresis and nailfold videocapillaroscopy	Preserved microvascular function with no differences in vasodilatory responses or capillary recruitment; no association with disease activity or inflammatory markers.
Foster et al., 2012 [41]	Cross-sectional	In total, 18 patients with early arthritis, 48 healthy controls and 25 disease controls	Laser Doppler Perfusion Imaging (LDPI)	Elevated endothelial injury markers in early inflammatory arthritis, suggesting early endothelial dysfunction independent of inflammatory burden.
Sandoo et al., 2011 [42]	Cross-sectional	In total, 99 patients with RA	Laser Doppler imaging + iontophoresis	Microvascular and macrovascular dysfunction appeared independent, without significant correlation.
Sandoo et al., 2012 [43]	Cross-sectional and longitudinal	In total, 99 RA patients in the cross-sectional and 23 RA patients starting anti-TNF-α treatment in the longitudinal	Laser Doppler imaging with iontophoresis	Endothelium-dependent microvascular function inversely associated with cardiovascular risk; transient improvement after anti-TNF-α therapy.
Sandoo et al., 2013 [44]	Cross-sectional	In total, 201 RA patients	Laser Doppler imaging with iontophoresis	CVD risk prediction scores were associated with microvascular and macrovascular function at the 6-year follow-up.
Klimek et al., 2017 [45]	Cross-sectional	In total, 75 patients with RA and ankylosing spondylitis (AS) and 26 healthy controls	Laser Doppler flowmetry	Altered microvascular function and reduced vasodilatory capacity in inflammatory arthritis; associations between inflammatory markers and microcirculatory parameters.
Ruiz-Limón et al., 2017 [46]	Longitudinal, interventional	In total, 20 RA patients, treated with tocilizumab for 6 months	Laser Doppler post-occlusive hyperemia	Improved endothelial-dependent microvascular function after tocilizumab; reduced oxidative stress and prothrombotic status.
Fenton et al., 2018 [47]	Cross-sectional	In total, 68 RA patients	Laser Doppler imaging with iontophoresis	Sedentary behavior negatively associated with endothelium-dependent microvascular function.
Dávida et al., 2020 [48]	Prospective, longitudinal	In total, 46 early RA patients and 8 early RA patients, treated with adalimumab	Laser Doppler flowmetry	PORH parameters correlated with macrovascular endothelial function; improvement after adalimumab treatment.
Anyfanti et al., 2023 [28]	Case–control, observational	In total, 35 RA patients (without CVD) and 35 controls	Laser speckle contrast imaging with reactive hyperemia	Impaired skin microvascular reactivity associated with myocardial microvascular perfusion and arterial stiffness independently of cardiovascular risk factors.

**Table 2:** Studies evaluating functional abnormalities of the dermal microvascular network using nailfold videocapillaroscopy in RA.

Study & Year of Publication	Study Design	Study Population	Method of Microvascular Assessment	Key Findings
Hachulla et al., 1994 [62]	Observational case–control study	A total of 9 patients with systemic rheumatoid vasculitis, 22 patients with active RA without vasculitis, and 16 healthy controls	Dynamic Nailfold fluorescence videomicroscopy	Increased capillary permeability was observed in RA patients both with and without vasculitis, indicating functional endothelial injury and altered capillary wall integrity.
Pache et al., 2002 [63]	Case–control study	In total, 12 RA patients and healthy controls	Nailfold capillaroscopy combined with cold provocation testing	Elevated levels of endothelin-1 associated with reduced capillary blood flow and cold-induced vascular dysregulation in RA.
Caraba et al., 2024 [64]	Retrospective, cohort study	A total of 70 RA patients and 70 matched healthy controls	Nailfold videocapillaroscopy with PORH and venous congestion protocols	RA patients presented lower capillary density at baseline, during PORH, and after venous congestion, suggesting impaired capillary recruitment and structural rarefaction. Capillary density improved after 12 months of anti-TNF-α therapy and was associated with DAS28 and TNF-α levels.

## Data Availability

The data presented in this study are available on request from the corresponding author.
